# Sea Buckthorn (*Hippophae rhamnoides* L.): Nutritional Significance, Phytochemistry, Molecular Mechanisms, Therapeutic Potential, and Emerging Applications in Food Systems

**DOI:** 10.3390/foods15081389

**Published:** 2026-04-16

**Authors:** Nazish Javaid, Adnan Amjad, Ralf Weiskirchen, Asad Abbas, Shehnshah Zafar, Mohibullah Shah, Muhammad Sameem Javed, Khurram Afzal, Umrah Zafar, Muhammad Israr

**Affiliations:** 1 Faculty of Food Science and Nutrition, Bahauddin Zakariya University, Multan 60800, Pakistan; nazishjavaid00@gmail.com (N.J.); shah8942123@gmail.com (S.Z.); sameemjaved@gmail.com (M.S.J.); khurram.afzal@bzu.edu.pk (K.A.); misrarjutt139@gmail.com (M.I.); 2 Institute of Molecular Pathobiochemistry, Experimental Gene Therapy and Clinical Chemistry (IFMPEGKC), RWTH University Hospital Aachen, D-52074 Aachen, Germany; 3 Department of Biochemistry, Bahauddin Zakariya University, Multan 60800, Pakistan; mohib@bzu.edu.pk; 4 Department of Human Nutrition and Dietetics, Muhammad Nawaz Shareef University of Agriculture, Multan 60000, Pakistan; umrah.zafar@mnsuam.edu.pk

**Keywords:** sea buckthorn, phytochemistry, therapeutic potential, food industry, encapsulation, industrial applications

## Abstract

Plant foods have been the cornerstone of human diets since ancient times, fueling civilization and shaping cultures. Plants became central to sustainable food systems, offering diverse and nutritious options for the future. Sea buckthorn (*Hippophae rhamnoides* L.) has attracted growing scientific interest due to the presence of bioactive compounds, polyphenols, fatty acids, phytosterols, carotenoids, vitamins, and minerals in its fruit, seeds, and leaves. Moreover, sea buckthorn exhibit antioxidant, anti-inflammatory, antimicrobial, antidiabetic, antihyperlipidemic, anticancer, hepatoprotective, neuroprotective, and metabolic regulatory properties supported by in vitro and in vivo models. The biological activity of these phytochemical compounds plays a crucial role in regulating the AMP-activated protein kinase (AMPK) and phosphoinositide 3-kinase/protein kinase B (PI3K/Akt) signaling pathways, as well as pro-inflammatory cytokines such as tumor necrosis factor-α (TNF-α) and interleukin-6 (IL-6), cell proliferation, and apoptosis. Furthermore, its potential against microbial growth, including *S. aureus*, *S. epidermidis*, *S. intermedius*, and *S. pyogenes*, among others, not only expands its applications in the pharmaceutical industry but also attracts researchers to incorporate it into food products. This could lead to the discovery of plant-based therapeutic products without significant adverse effects. However, further exploration of each component’s potential side effects is necessary to support the commercialization of formulated products in either the pharmaceutical or food industries, ensuring the highest safety standards for consumers. Including studies on bioavailability and pharmacodynamics could further strengthen the scientific evidence supporting the specific phytochemicals in sea buckthorn and their mechanistic interactions.

## 1. Introduction

The sedentary lifestyle, poor diet (high in refined carbohydrates, sugary beverages, saturated/trans fats), existential distress/anxiety, sleep deprivation, and smoking all contribute to several health risks [1]. Demographics such as aging, ethnicity (particularly, Asian, American, Pacific Islander, African American, and Hispanic/Latino), and historical antecedents are associated with health-related issues [2]. Furthermore, vital organs like the heart, kidneys, brain, and liver globally show defects in response to lifestyle changes [3]. Metabolic disorders such as diabetes, hypertension, and polycystic ovary syndrome (PCOS) present major challenges that burden the global health system [4]. While drugs have supported the health system in addressing these burdens, their adverse effects remain a concern for medical professionals [5].

This has prompted researchers and clinicians to find alternative ways to support pharmaceutical or adjuvant therapies [6]. Previous studies have shown that plants naturally contain bioactive compounds that play a key role in regulating biological functions and reducing the burden of ailments [7]. These plants possess a unique nutritional profile and bioactive ingredients crucial in regulating different pathways to prevent, treat, and manage various diseases [8]. *Hippophae rhamnoides* L., commonly known as sea buckthorn (SB), dry spine or vinegar weed, is a perennial deciduous shrub of the family *Elaeagnaceae* and has been widely used for its therapeutic potential in addressing various health complications. Bioactive substances like flavonoids and palmitoleic acid found in SB enhance biological activity by modulating signaling pathways of AMPK and PI3K/Akt [9].

SB is a rich source of antioxidants and bioactive compounds (polyphenols, carotenoids, vitamins, fatty acids, phytosterols, polysaccharides, and other secondary metabolites). Experimental studies have shown that SB provides a variety of biological and physiological effects. Preclinical evidence indicates that SB may exhibit immune-modulating and anti-oxidative properties [10], cardio-protective and anti-atherogenic effects [11,12], as well as antiviral and antibacterial activities [13]. Additional studies in vitro and in animal models suggest potential wound-healing [14], anti-inflammatory [15], antidiabetic [16], anticancer [17], and hepatoprotective or dermatological benefits [13,18]. This versatility has led to the extensive use of SB, whether in the form of berries, leaves, or seeds as a therapeutic or nutraceutical product. Clinical studies have indicated that SB supplementation can help decrease fasting plasma glucose levels in individuals with impaired glucose regulation [19], suggesting its potential as a complementary approach to dietary management in diabetes care [20].

Several recent reviews have summarized the nutritional composition, bioactive compounds, and general health effects of SB, including its role in metabolic disorders and its use in functional foods [21,22,23,24,25,26]. However, there is still a need for an integrative, part-specific synthesis that (i) systematically compares the macronutrients, micronutrients, and phytochemical profiles of all major anatomical parts (berries, pulp oil, seeds, leaves, twigs, branches) including often under-utilized biomass streams, (ii) maps individual or grouped SB phytochemicals to well-characterized molecular targets and signaling pathways (e.g., AMPK/SIRT1, PI3K/Akt, Nrf2, NF-κB, PPARγ/LXRα-ABCA1/ABCG1, TGR5-UCP1/PGC1α, ERK/CREB/BDNF) across multiple organ systems, and (iii) links this mechanistic evidence directly to emerging encapsulation technologies and food-system applications, with a focus on stability, bioaccessibility, and circular-economy-oriented valorization of co-products. The present review addresses these gaps by (1) providing a detailed, quantitative comparison of nutrients and bioactive compounds in different SB parts, (2) critically synthesizing preclinical and clinical evidence around defined molecular pathways involved in metabolic, cardiovascular, hepatic, neurodegenerative, dermatological, and respiratory disorders, and (3) summarizing recent advances in encapsulation and food processing that enable the incorporation of SB ingredients into diverse food matrices while preserving or enhancing bioactivity.

Thus, this review not only summarizes the nutritional composition and phytochemistry of SB but also emphasizes mechanistic links between specific phytochemicals and signaling pathways, integrates current preclinical and clinical evidence, and highlights emerging encapsulation strategies and food applications that can translate these bioactivities into safe, functional products.

## 2. Search Methodology

### 2.1. Search Strategy and Literature Identification

A literature review was conducted to gather studies on the nutritional composition, phytochemistry, molecular mechanisms, therapeutic potential, and food applications of *Hippophae* species. Major scientific databases, including Scopus, Web of Science, PubMed, and Google Scholar, were searched to identify relevant peer-reviewed articles, review papers, and book chapters. The search utilized the following keyword combinations: “sea buckthorn,” “*Hippophae*,” “nutritional composition,” “bioactive compounds,” “polyphenols,” “phytochemistry,” “antioxidant activity,” “molecular mechanisms,” “therapeutic properties,” and “functional food applications.”, applied individually and in Boolean combination. Publications from 2000 to 2024 were primarily considered to capture recent developments, while earlier key studies were included where necessary to provide background and foundational information.

### 2.2. Study Selection and Data Synthesis

After the initial search, the accessed articles were screened for relevance to the objectives of the review. Studies addressing nutritional composition, bioactive phytochemicals, pharmacological activities, molecular pathways, and food applications of SB were prioritized. Duplicate records and studies lacking sufficient scientific evidence or methodological clarity were excluded. The selected full-text articles were examined to extract information on phytochemical constituents, biological mechanisms, therapeutic effects, and applications in functional foods and nutraceutical products. The findings were then synthesized through critical evaluation of the methodologies and results reported in the selected studies to provide a comprehensive overview of the nutritional and functional importance of *Hippophae* species.

## 3. Morphology and Botanical Descriptions

**Figure 1:**
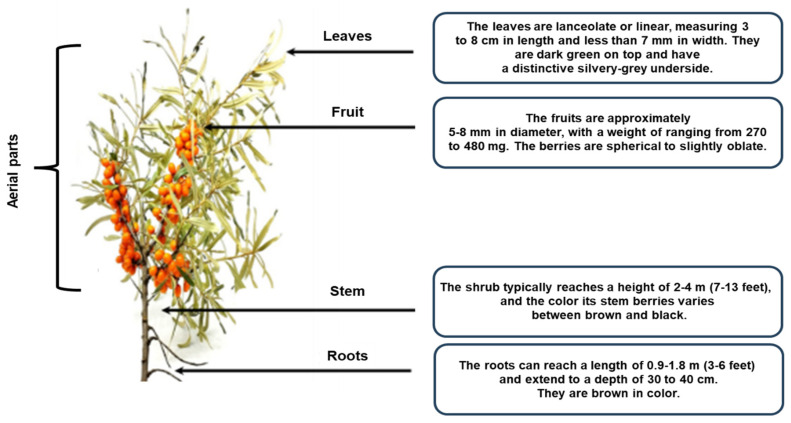
Morphological characteristics of sea buckthorn (*Hippophae rhamnoides* L.). The figure displays the main plant parts, such as aerial organs (leaves, fruits, stems) and roots. The leaves are lanceolate to linear, with a dark green upper surface and silvery-gray underside. The fruits are spherical to slightly oblate, measuring 5–8 mm in diameter. The shrubs usually grow to a height of 2–4 m, with roots extending 0.9–1.8 m in length to a depth of 30–40 cm.

The SB belongs to the genus *Hippophae* L. and the family *Elaeagnaceae*. Some parts of the SB can reach up to 18 m in height, but it typically ranges from 1 to 8 m in height. The leaves are lanceolate or linear, usually 3–8 cm long and less than 7 mm wide [27]. It is a deciduous shrub of the *Elaeagnaceae* family, reaching a height of 2–6 m (Figure 1). The upper surface of the SB leaves is dark gray, while the lower surface is distinctively silver-gray [28]. The fruits have a diameter of 5–8 mm, are round or oblate, and several fruits are clustered together [29]. The fruit of the SB is brownish-red or orange-yellow in color with a rough surface. The pulp of the SB is oily and soft in texture. The seeds of the SB are obliquely ovate, approximately 4 mm long and 2 mm wide, shiny and brown in the middle with a longitudinal channel. The seed coat and seed kernel of the SB are hard and creamy white. SB, as an ecologically and economically valuable plant, has garnered significant interest due to its abundant natural bioactive components. The presence of bioactive compounds in the berries and leaves makes them a valuable medicinal and edible plant resource [30].

## 4. Nutritional Composition and Phytochemistry of Sea Buckthorn Parts

Across its different anatomical parts, SB provides a dense matrix of macronutrients, micronutrients, minerals, and specialized phytochemicals that collectively underpin its nutritional and functional relevance (Table 1).

### 4.1. Fruit (Pulp)

The berries of SB are incredibly nutritious and often referred to as a ‘super fruit’. The bioactive components of SB berries depend on the fruit’s maturity, size, geographical location, and extraction methods [41]. SB fruit is rich in carotenoids (such as β-carotene, zeaxanthin, lycopene), vitamins (such as vitamin C), phytosterols (like β-sitosterol, making up 57–83% of total sterols), sugars (such as fructose, glucose), organic acids (such as malic, quinic), minerals (such as potassium, manganese, copper), fiber, amino acids, and polyphenols [33]. The SB pulp is particularly abundant in monounsaturated (palmitoleic) and saturated (palmitic) fats, carotenoids (up to 1000 mg/100 g in pulp oil), tocopherols, and plant sterols [42]. The pulp is widely used in cosmetics and nutraceuticals for its rich pigmentation, antioxidant profile, and hydrating properties. It is commonly found in dietary supplements, skin care products, and serums [43].

### 4.2. Seed

Although the seeds are botanically contained within the SB berry, they are considered separately in this review because seed oil and seed meal differ significantly from the pulp in terms of fatty acid profile, micronutrient content, and technological applications. SB seeds are a highly valuable component of the plant recognized for their rich nutritional and bioactive composition. The seeds of SB contain a significant amount of oil (approximately 8–18%), characterized by a balanced profile of polyunsaturated fatty acids, particularly omega-3 (α-linolenic acid) and omega-6 (linoleic acid) [33,44]. SB seeds are also a good source of protein (20–25%) and dietary fiber. These seeds are effective in treating burns, ulcers, psoriasis, eczema, and supporting wound granulation. It is also used to manage gastric ulcers, inflammation, cervical/vaginal erosion, and to soothe pyretic and viral conditions [45]. The cardiovascular and metabolic benefits include lowering cholesterol, supporting circulation, having anti-atherogenic action, and reducing the risk of thrombophlebitis [46].

### 4.3. Leaves

SB leaves contain bioactive compounds, such as flavonoids like isorhamnetin, kaempferol, quercetin or glycosides, tannins (Gallo-/ellagitannins), anthocyanins, and alkaloids [38]. Flavanols and leucoanthocyanidins are characteristic polyphenolic compounds found in SB leaves [47]. The leaves are a rich source of vital antioxidants, including carotene, vitamin E, catechins, ellagic acid, ferulic acid, folic acid, as well as significant amounts of magnesium, potassium, and calcium [39].

### 4.4. Twigs

**Figure 2:**
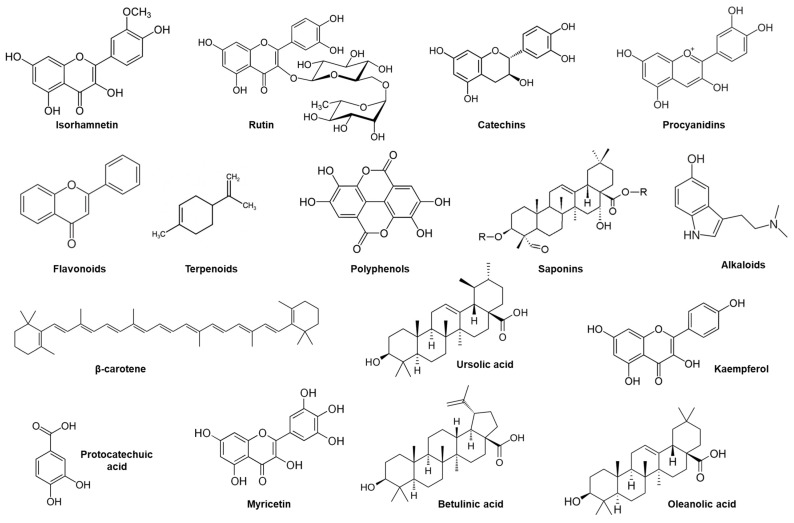
Representative phytochemicals identified in different anatomical parts of sea buckthorn (*Hippophae rhamnoides* L.), including berries, leaves, and twigs, along with their chemical structures, highlighting phenolic compounds (e.g., flavonoids, procyanidins) that are particularly abundant in twig and leaf extracts. The image displays specific phenolic compounds and representatives of compound classes.

SB twig extracts contain a remarkably high level of phenolic compounds. Figure 2 illustrates representative phytochemicals from various SB parts, with a specific emphasis on phenolics found in twigs and leaves. Butanol-based extracts can reach up to 621 mg/g of dry extract, which is higher than leaf extracts (341.5 mg/g) [48]. The twigs of SB are also rich in catechins and procyanidins. Twigs or branches of SB plants were often discarded [40]. In terms of vitamins, only α-tocopherol (vitamin E) was identified in twig extracts (Table 1). Twigs also contain a notable amount of essential amino acids, with arginine being the most prevalent at 0.87 g/100 g of dry material [48]. The overall proportion of essential amino acids in twigs is comparable to that of leaves, accounting for approximately 63%. These compounds have potential uses in medicine and cosmetics. Recent studies have shown that they contain bioactive antioxidants and anticoagulants [49].

## 5. Medicinal Attributes and Bioactivities of Sea Buckthorn

### 5.1. Antidiabetic Activity

Diabetes mellitus has become more prevalent in recent decades, affecting an estimated 463 million adults. According to the International Diabetes Federation, the global number of people with diabetes reached 537 million in 2021, and this figure is expected to rise to 783 million by 2045 without major interventions [50]. Recent studies have shown that dietary interventions encourage lifestyle modifications as a suitable substitute to regulate glucose homeostasis [51,52]. SB may help prevent diabetes by regulating insulin secretion and glucose metabolism through stimulating the coupled receptors of G protein. The SB pulp oil, high in palmitoleic acid, an exceptional fatty acid present in plants, may improve the production of glucose-induced insulin in human islet cells. SB seed protein can improve insulin sensitivity, decrease oral glucose tolerance, modulate hepatic glucose metabolism genes, and the AMPK/SIRT1 pathway in diabetic mice [53]. SB oil and extract can stimulate glucose transporter 4 (GLUT4) translocation, enhancing glucose uptake into cells, mimicking insulin’s action which leads to a 10% reduction in circulating glucose in a chick embryo model, and a full reversal of hyperglycemia in *Caenorhabditis elegans* [54].

SB demonstrates antidiabetic potential by enhancing glucose-stimulated insulin secretion, likely through activation of G protein-coupled receptor pathways by palmitoleic acid-rich pulp oil [55]. Additionally, SB seed protein improves insulin sensitivity and reduces insulin resistance via modulation of hepatic glucose metabolism and activation of the AMPK/SIRT1 signaling pathway in diabetic models [56].

SB oil and extracts have been reported to stimulate GLUT4 translocation, thereby enhancing cellular glucose uptake in an insulin-like manner. However, these findings are primarily derived from non-mammalian experimental models, including chick embryo and *Caenorhabditis elegans*, where modest reductions in circulating glucose and reversal of hyperglycemia were observed [57]. Other findings report that both hyperglycemia and excessive water intake reduced sorbitol accumulation in the eye lens, a sign of improved glucose metabolism [44]. In another study on metabolic syndrome, the ability of SB to improve glucose metabolism, inhibit β-glucosidase (limiting carbohydrate digestion), alleviate hypertension, and protect the cardiovascular system was highlighted, demonstrating its potential as both preventive and therapeutic for metabolic disorders, including diabetes [58]. Similarly, the antidiabetic effect of SB was demonstrated in rats when fed at doses of 100, 200, or 300 mg/kg/day for 4 weeks [59]. The extract of SB fruit oil demonstrated significant metabolic regulation by enhancing glucose uptake in insulin-resistant HepG2 cells, increasing concentrations from 12.23 ± 1.09 to 14.90 ± 1.48 mmol/L. This cellular improvement translated to systemic benefits in vivo, where SBFO dosages of 200 and 300 mg/kg/day achieved blood sugar reduction rates of 10.47% and 13.79%, respectively. Clinically, these findings suggest that SBFO may act as an insulin sensitizer, potentially mitigating the hyperglycemia associated with type 2 diabetes by restoring glucose homeostasis and improving insulin indices [60].

**Figure 3:**
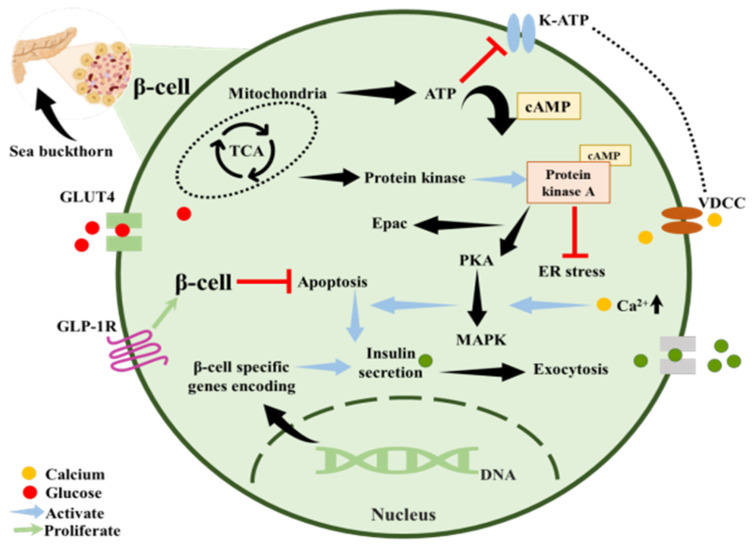
Mechanism of sea buckthorn activity. Shown is the regulation of insulin secretion in β-cells, with a focus on the interaction between mitochondrial adenosine triphosphate (ATP) production, cyclic adenosine monophosphate (cAMP) signaling, and downstream pathways like protein kinase A (PKA), exchange protein directly activated by cAMP (Epac), and mitogen-activated protein kinase (MAPK). Increased ATP levels inhibit specific channels, enhancing Ca^2+^ influx, which triggers the exocytosis of insulin-containing vesicles. At the same time, PKA and MAPK affect β-cell gene expression, reduce apoptosis, and suppress endoplasmic reticulum stress to maintain cell function. This mechanism demonstrates how metabolic and signaling pathways collaborate to enhance insulin secretion and β-cell survival. It is important to note that most of the evidence supporting these mechanisms comes from in vitro or animal models, and further validation is required to apply these findings to human physiology.

In another meta-analysis, it was found that SB supplementation significantly lowered blood pressure and blood sugar in overall subjects with metabolic syndrome, indicating that its glucose-lowering effects might be context-dependent [22]. The presence of polyphenol extract in SB shows promising anti-obesity and antidiabetic potential [61]. Figure 3 depicts how different pathways are associated with the antidiabetic effect of SB.

### 5.2. Anti-Inflammatory Activity

The SB is rich in bioactive substances with potent anti-inflammatory properties, including carotenoids, flavonoids, and fatty acids that exhibit strong anti-inflammatory effects. These phytochemicals regulate pro-inflammatory cytokines (e.g., TNF-α, IL-6) and inhibit the oxidative stress pathway [62]. The use of SB in traditional Chinese medicine has been a major way of curing various serious diseases [63]. SB provides high levels of polyphenols and flavonoids, which offer robust antioxidant protection and anti-inflammatory activity [64]. The extracts from various sections of the fruit (pulp, peel, seed) and the peel extract were revealed to have the highest reducing edema\activity. The abundance of omega-3 fatty acids in seed oil gives it anti-inflammatory and insulin-sensitizing properties [37].

Similarly, another study showed that the berry extract of SB fed to mice enhanced phase II and antioxidant enzyme activity in the liver and decreased oxidation activities in the cellular location. The anti-inflammatory properties in SB have been demonstrated in several in vivo studies [65]. SB cultivars containing high oil and flavonoid contents have a higher economic value, which are further increased by specialized enzymes within the plants [66]. The occurrence of mitogens in SB provokes lymphocyte proliferation. SB interacts with C-reactive protein, which is a marker of inflammation and a risk factor [67]. The leaf extracts kill harmful bacteria like MRSA and *S. aureus*, fight against viruses like herpes simplex virus type 2 (HSV-2), and help vaccines work better by boosting the immune response [68]. The neuroprotective activity of SB removes the amyloid-beta (Aβ) deposits from the brain, reduces brain inflammation, and protects the brain from Alzheimer- like damage in animal studies [69].

Other findings have revealed that supplementation reduces pro-inflammatory cytokines (IL-1, IL-2), upregulates anti-inflammatory IL-10 regulation, and downregulates TLR4 and NF-κB pathways to improve the integrity of gut barriers and health [70]. Similarly, in other studies it was found that the fruit extract of SB at concentrations of 5–100 μg/mL inhibited the secretion of cytokines (IL-6, TNF-α, IL-1β) and stimulated human neutrophils and macrophages, showing dose-dependent anti-inflammatory activity [71]. Another study by Xiao et al. (2021) demonstrates that in a mouse model of high-fat diet-induced hyperlipidemia, supplementation with SB oil significantly reduces inflammation and liver steatosis through modulation of the peroxisome proliferation-activated receptor-γ (PPARγ)-liver X receptor α (LXRα)-ATP-binding cassette transporter A1 (ABCA1)/ATP-binding cassette transporter G1 (ABCG1) pathways [72]. Similarly, a human randomized double-blind placebo-controlled trial reported that healthy adults who consumed 28 g/day of SB berry puree for 90 days showed a small but significant reduction in serum C-reactive protein, a key inflammation marker [73].

### 5.3. Anti-Hyperlipidemia Activity

Hyperlipidemia, characterized by abnormal lipid metabolism and fat transport problems, is a metabolic and endocrine disorder [74]. According to recent global trends, the age-adjusted prevalence of hypercholesterolemia may have peaked in certain areas, but the number of affected individuals continues to rise due to population growth and aging, especially in low and middle-income countries. Phytosterols, bioactive compounds found in the lipids of SB pulp, play a crucial role in preventing cardiovascular diseases (CVDs), particularly hypercholesterolemia [75]. The phytosterols have a greater cholesterol-lowering effect, reducing low-density lipoprotein cholesterol (LDL-C) levels by approximately 10 to 15% and inhibiting endogenous cholesterol reabsorption and the stimulation of endogenous cholesterol reabsorption as a neutral steroid [76].

Previous studies have demonstrated the in vivo anti-hyperlipidemic effects of SB through animal trials. SB seed extract, enriched with flavonoids at doses of 100 and 300 mg/kg, reduced liver triglyceride levels in obese mice by 16.67% or 49.56%, respectively. Follicle-stimulating hormone (FSH) can enhance lipid metabolism by inhibiting PPARγ, enhancing PPAR alpha expression, and suppressing adipose tissue [77]. Recent studies involving a clinical trial with 19 participants, aged 54.06 ± 2.97 years, who consumed 50 mL of SB juice daily for eight weeks, showed significant reductions in LDL-C and body/visceral fat, along with an increase in HDL-C, C-reactive protein (CRP), and certain immunoglobulins, while triglyceride levels remained unchanged [78].

Other studies have examined the impact of consuming SB puree on reducing blood cholesterol and other risk factors of CVD. In a study, 111 patients with hypercholesterolemia were given 90 mL of SB puree or a placebo for 90 days. The results showed no significant changes in total cholesterol, LDL-C, or triglycerides [74]. HDL-C initially decreased in the first 45 days but then increased in the last 45 days (*p* < 0.05), along with a slight decrease in diastolic blood pressure and high-sensitivity C-reactive protein (hsCRP). More research is needed to determine the potential benefits and key sources of SB bioactive compounds for the treatment and prevention of cardiovascular disorders. Previous studies have also suggested that AMPK can activate the PI3K/Akt signaling pathway, leading to the inactivation of a specific isoform of glycogen synthase kinase 3 beta (GSK3β), enhancing GS expression, and regulating glucose transport activity [79]. Another study in golden Syrian hamsters using SBFO extracts at doses of 50, 100, and 200 mg/kg/day investigated their role in preventing hyperlipidemia. At the molecular level, the anti-hyperlipidemia effectiveness of SBFO through the AMPK and Akt signaling pathway was examined using RT-PCR and Western blot analysis [80].

### 5.4. Antioxidant Activity

Antioxidants are substances that help protect cells from damage caused by free radicals, which are unstable compounds that can lead to diseases such as CVD, cancer, and stroke [81]. The SB market is projected to grow at an average annual rate of 7.3% to reach US $495.9 million by 2030 from an estimated US $324.1 million in 2024. Studies have shown that oil produced from berries and pulp of SB has antioxidant activity in various models. Additionally, SB can regulate nuclear factor erythroid 2-related factor 2 (Nrf2), a transcription factor that promotes the formation of antioxidant enzymes, such as glutathione peroxidase and catalase [82]. SB oil stimulates mechanisms of apoptotic cell cycle and autophagy, removing damaged or abnormal cells and halting the growth of many cancer cells.

A clinical trial investigated the effects of consuming 50 mL of SB juice daily for 8 weeks in women with hypercholesterolemia [78]. The findings indicated significantly increased antioxidant markers, improved lipid profile, and reduced oxidation of low-density lipoprotein (LDL). Another human study demonstrated that consuming SB juice provided additional flavonoids (355 mg/day) along with β-carotene, α-tocopherol, and vitamin C, enhancing the antioxidant capacity of plasma over 8 weeks in healthy male volunteers [83]. Similarly, a study on aging-related animal and cell models found that an ethanolic extract from SB seed residues protected murine cells against oxidative stress-induced damage and apoptosis [84].

### 5.5. Anti-Obesity Effects

**Figure 4:**
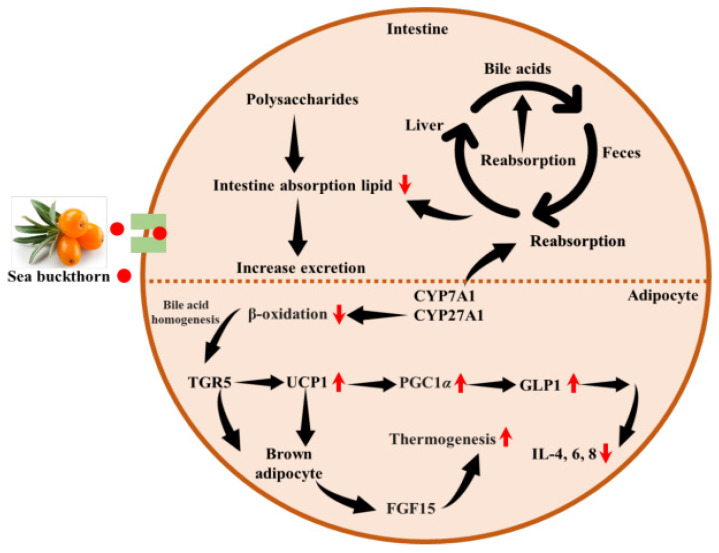
Anti-obesity mechanisms of sea buckthorn, highlighting effects on bile acid circulation, lipid absorption, thermogenesis, GLP-1 secretion, and inflammatory cytokines via pathways including CYP7A1/CYP27A1 and TGR5 signaling. Abbreviations used are: CYP7A1/CYP27A1, Cholesterol 7-alpha-hydroxylase; FGF15, fibroblast growth factor 15; GLP1, Glucagon-Like Peptide-1; IL-4, 6, 8, interleukin-4, -6, -8; PGC1α, Peroxisome Proliferator-Activated Receptor Gamma Coactivator 1-α; TGR5; Takeda G Protein-Coupled Receptor 5, UCP1, Uncoupling Protein 1.

Obesity is often linked to an excess of body fat. Studies have shown that children born to obese mothers are more prone to developing health issues related to obesity, metabolic syndrome, or vascular disease [85]. Recent investigations have identified that maternal obesity can impact the epigenetics of adipose tissue, as well as alter nutritional metabolism and endocrine function [86]. Adipose tissue, composed of specialized fat-managing cells called adipocytes, is a dynamic organ that expands continuously during obesity [87]. Studies have shown that the fruit oil of SB can reduce obesity caused by hyperlipidemia at doses of 100 and 300 mg/kg, significantly decreasing the body weight (BW) in obese mice fed a high-fat diet by 33.06% or 43.51%, respectively [75]. Another study [88] demonstrated a novel approach that increases pyruvate kinase M2 (PKM2) and fatty acid synthase (FASN) expression during SB dietary intervention in pregnant rats to reduce the weight and body fat of offspring. The results showed a decrease in key inflammatory factors such as TNF-α and IL-6, as well as improved energy metabolism (Figure 4).

Others investigated the anti-obesity effect of polysaccharide fractions that inhibited lipid accumulation by enhancing PPARγ, coactivator 1 alpha (PGC1α), PR domain-containing protein 16 (PRDM16), and uncoupling protein-1 (UCP-1) [89]. The regulation of these expressions improves thermogenesis by activating brown adipocytes, leading to lower BW. Previously, studies indicated that rats fed SB leaf tea at doses of 1% and 5% by weight significantly restricted weight gain by modulating phosphatidate phosphatase (PAP), carnitine palmitoyltransferase (CPT) activities, and β-oxidation [90]. The simultaneous enhancement of glucose-6-phosphate dehydrogenase (G6PD) and decrease in erythrocyte lipid peroxides in relation to oxidative markers such as superoxide dismutase (SOD), catalase (CAT), and glutathione peroxidase (GPx), ultimately help maintain normal weight. Additionally, SB powder feeding has shown reliable outcomes in reducing or modulating weight gain, fat mass, and lipid circulation in rats fed a high fat diet. This is achieved through the regulation of cytokines such as IL-4, IL-6, IL-8, interferon-γ, and TNF-α in both white and brown adipose tissue [91]. Similarly, the bioactive chemical profile of SB has been demonstrated to have a natural ability to reduce weight gain through its anti-oxidant and anti-inflammatory properties [70,92].

### 5.6. Anticancer Effects

Cancer is one of the leading causes of death worldwide. Nearly 10 million people died from cancer in 2020, with almost 20 million new cases and 9.7 million cancer-related deaths reported globally in 2022. Cancer is a complex illness characterized by uncontrolled cell proliferation resulting from genetic mutations and environmental factors such as smoking or exposure to carcinogens. Cancer treatment usually involves radiotherapy or chemotherapy, but it is often associated with common side effects like hair loss, nausea, or sore throat [93]. A study investigated the anticancer potential of methanolic SB root extract against head and neck cancer cell lines using LC/MS and NMR for structural analysis. The findings of this study revealed that the aqueous phase of the extract reduced the cancer cell viability while leaving non-tumorigenic controls cells unaffected [94].

SB pulp oil, high in palmitoleic acid, may activate caspase-3 and caspase-9 pathways to stop leukemia cells from proliferating and induce apoptosis. SB seed oil alters the expression of genes p53, Bax, and Bcl-2, due to the presence of omega-3, 6, 7, and 9 fatty acids [23]. These fatty acids inhibit the growth of human breast cancer cells and enhance cytotoxicity. Extracts from sea berries, rich in phenolic compounds like flavonoids, may help rats avoid colon cancer by reducing bile acid levels in feces and enhancing detoxifying enzyme activity. In animal modeling, SB leaves demonstrated anticancer potential in the lungs by exhibiting anti-metastatic and anti-angiogenic actions through inhibition of metalloproteinases and vascular endothelial growth factor [80].

Similarly, Fu and colleagues reported the protective effect of concentrated/fermented SB compound juice against colorectal cancer, when given orally in preclinical colorectal models [95]. It reduced tumor growth, reshaped the gut microbiome, and improved antitumor immunity. Another study showed that iso-rhamnetin induced apoptosis, cell cycle arrest, and reduced metastatic traits in breast, colorectal, and liver cancer lines at around 10–100 µM (approximately 3–30 µg/mL) and showed synergy with some chemotherapeutics in selected models [96]. Similarly, it was demonstrated that the SB oil protected against chemotherapy-induced organ toxicity and, in some models, reduced tumor progression when given as an adjunct (oral oil doses in animals often reported as 100–1000 mg/kg depending on study formulation) [97,98]. Other findings revealed that extract and isolated flavonoids (quercetin and isorhamnetin) inhibited proliferation and induced apoptosis in breast cancer in small animal studies, with effective ranges commonly around 25–150 µg/mL, resulting in reduced tumor expansion [99]. Another study suggests that SB leaf extracts enhance cytotoxicity against cancer cell lines at low µg/mL ranges in plant-mediated selenium nanoparticles, indicating that nanoformulations may boost anticancer potency [100]. Furthermore, it was shown that aqueous fractions tested at 50–200 µg/mL in vitro were characterized through chemical profiling. The aqueous fraction of the methanolic root extract of SB exhibited strong cytotoxicity against head and neck cancer cells [94].

### 5.7. Anti-Asthmatic Activity

Asthma is characterized by chronic airway inflammation caused by infiltrating eosinophils, T lymphocytes, and mast cells, or the release of pro-inflammatory cytokines and lipid mediators [101]. According to the Global Initiative for Asthma, by 2025, there may be 400 million cases of asthma [102]. SB oil, produced from the berries’ seed and pulp, is used as a medication to clear the lungs and loosen phlegm, which can reduce wheezing and coughing, two common asthmatic symptoms. Additionally, the antioxidant and anti-inflammatory effects of SB oil may protect the cells and tissues of the respiratory system from inflammation and oxidative stress (Table 2).

Asthma attacks, triggered by allergens, irritants, or infections, can be prevented or reduced with the help of SB oil. SB’s berries and leaves are also edible and used as supplements or food. They contain a variety of bioactive substances that may be therapeutic to the respiratory system, including fatty acids, carotenoids, phenolic acids, and flavonoids. SB leaves and berries may have anti-diabetic, neuroprotective, anti-lipoxygenase, anti-obesity, and anti-lipoxygenase effects that can help with mood, blood sugar, BW, and inflammatory levels, all of which can impact asthma, according to some research [103].

**Table 2:** Anti-asthmatic effect of sea buckthorn.

Ingredients/Components	Model/Formulation and Reported Dose	Findings	Refs.
SB flavonoid extract (SBF and PSBF)	25–100 µg/mL (mast cells)	Inhibition of IgE-mediated mast cell degranulation and MAPK(p38/JNK) signaling	[104]
Polyphenols/isorhamnetin, chemical mediator assays	2–20 µM (cell models)	Reduction of histamine and leukotriene release, leading to an anti-allergic effect	[105]
Total flavonoids from SB (TFSB) in airway inflammation models	µg/mL (in vitro and animal models)	Decreased airway inflammation and cytokines (IL-1β, IL-6) in bronchial epithelial cells stimulated by LPS/CSE in rodents	[106]
Isorhamnetin in cigarette smoke and COPD mouse models	Low µM (in vitro/in vivo)	Activation of the Nrf2 pathway, reducing oxidative stress and inflammation	[107]
Ethanolic extract of *H. rhamnoides* in RDS and acute lung injury (rat)	Animal models (oral/intraperitoneal)	Reduction of lung injury, edema, and inflammation	[108]
SB formulations in COPD and airway disease models	Animal studies	Improvement of lung function, reduction of airway inflammation, modulation of the gut–lung axis, and antioxidant effects (NOX4/ferroptosis, p53/MAPK pathways in COPD models)	[109]

### 5.8. Anti-Microbial/Anti-Viral Activity

Antimicrobial resistance is a significant global health concern, resulting from the inappropriate and excessive use of antibiotics, leading to the emergence of multidrug-resistant pathogenic microorganisms [110]. Bacterial infections are estimated to cause 7.7 million deaths annually, with approximately 4.95 million linked to drug-resistant pathogens, including 1.27 million deaths directly caused by organisms resistant to current antibiotics [111]. SB contains various bioactive compounds and nutrients such as vitamins, carotenoids, and polyphenols. Studies have shown that SB exhibits anti-microbial and anti-viral activities in vitro. It was found that all 67 g-positive bacteria obtained from clinical samples were considerably inhibited by the SB leaf extract [112]. At a 5% concentration, the extract inhibited nearly 50% growth of *S. aureus*, *S. epidermidis*, *S. intermedius*, and *S. pyogenes*. Additionally, SB extracts can mitigate the adverse effects of S. aureus on human keratinocytes by inhibiting apoptotic and pro-inflammatory cytokine pathways, including matrix metalloproteinases (MMPs), caspases, transforming growth factors (TGFs), IFNs, and fibroblast growth factors (FGFs) [113].

Similarly, it was shown that the 6 mg/mL extract of SB berries and leaves inhibits the growth of *S. aureus* bacteria that are resistant to methicillin [114]. Others found that the mouthwash successfully created with SB pulp oil demonstrated bactericidal effects on some pathogenic periodontal properties, stopping the production of single multi-strain biofilms [112]. In another study, the addition of 4% SB leaf to honey produced a preparation with a broad activity spectrum, with reported minimum inhibitory concentration against some strains being around 250 mg/mL in that formulation, and the mix inhibited biofilm formation in tested bacteria [115].

Likewise, others evaluated the antimicrobial activity of hydroalcoholic leaf extract of SB against different bacteria, including *Pseudomonas aeruginosa*, *S. aureus*, and *Enterococcus faecalis* [116]. The findings showed clear inhibitory zones for *Pseudomonas aeruginosa*, *S. aureus*, and *Enterococcus faecalis* and indicated the potency of tannin/flavonoid content. In another study, significant antimicrobial activity of fermented juice was found, showing stronger inhibition of *E. coli*, *S. aureus*, and fungal strains compared to unfermented juice [13]. The results of the study demonstrated that fermentation increased total flavonoids and enhanced the antibacterial and antifungal effects.

The anti-biofilm and antibacterial action of SB leaf enriched with 4% honey showed broad-spectrum activity, with reported minimum inhibitory concentration (MIC) for the best formulation at approximately 250 mg/mL in the honey matrix [115]. The outcomes of the research indicate that the combination inhibited biofilm formation and had broader antimicrobial coverage than honey alone. In another study, it was demonstrated that the purified phenolic-free fraction reported MIC of approximately 0.25 mg/mL for *S. aureus* and 0.5 mg/mL for other bacteria [114]. The findings of the study indicated that active phenolics concentrated fractionation significantly increases the antimicrobial activity versus crude extract, showing that the targeted phenolic concentrates are more effective. Likewise, a study assessed the decontamination impact of the extract following a 15-min seed treatment. The results showed that the extract was more effective against *Salmonella enterica*, *E. coli*, and *S. aureus* than active chlorine. The decontamination rates ranged from 72.1% for *Staphylococcus aureus* to 93.0% for *Listeria monocytogenes* [117]. *S. aureus* showed the highest decontamination rate at 81.9% after soaking seeds in the extract for 3 h.

### 5.9. Hepatoprotective Activity

Hepatotoxicity occurs when toxins weaken the liver’s antioxidant defenses, leading to oxidative stress, inflammation, and hepatocyte destruction [118]. Common causes include hepatic ischemia–reperfusion injury, drug-induced liver injury (DILI), alcoholic liver disease (ALD), and non-alcoholic fatty liver disease (NAFLD). Globally, liver diseases affect more than 25% of the population, representing a significant health burden and contributing to significant healthcare costs [119]. Studies on SB extracts, including flavonoids, carotenoids, and sterols, have shown hepatoprotective effects. They reduce liver enzymes, such as alanine aminotransferase (ALT) and aspartate aminotransferase (AST), improve lipid profiles (HDL, LDL), and attenuate liver fibrosis and inflammation in animal models (e.g., NAFLD mice, bile duct ligation) and in limited human trials. SB’s various bioactive compounds can reduce levels of liver enzymes that are markers of liver injury, such as ALT and AST. Hepatic fibrosis, or the scarring of liver tissue caused by ongoing inflammation or injury, can be attenuated or reduced. Recent research indicates that BALB/c mice were administered oral SB berry seed oil (40 mg/kg BW) and cyclophosphamide (25 mg/kg) for 10 days, which caused liver damage. Findings from Saeed and colleagues showed that the co-administration of SB berry seed oil (SBO) significantly reduced the increase in sinusoidal injury and hepatic injury biomarkers, suggesting its hepatoprotective benefits [120]. Another study demonstrated beneficial effects in rats pretreated with the leaf extract of SB at doses of 50, 100, and 200 mg/kg BW for 5 days before inducing liver injury with carbon tetrachloride. The findings of the study reported that all doses significantly reduced the liver enzyme levels and improved the histopathological activities, with the 200 mg/kg dose showing the most effective results [121].

Another study found that the active ingredients in SB inhibit the activation of hepatic stellate cells, attenuate liver fibrosis in the bile duct ligation model after appropriate challenge, and lower inflammatory cytokine levels [122]. The findings of a recent study suggest that all doses significantly reduce liver enzyme levels, malondialdehyde content, and activities of antioxidant enzymes, with the 0.26 mg/kg dose showing the most significant effects [123]. HepG2 cells were treated with 50 µg/mL SB leaf ethanol extracts. The findings suggest that the treatment significantly reduced the expression levels of hepatitis B surface antigen (HBsAg) and hepatitis B e antigen (HBeAg), with quercetin and kaempferol identified as the active constituents responsible for the anti-hepatitis B virus (HBV) effects [98]. A study by Liu confirmed the beneficial effects during acute liver failure in mice [124]. Likewise, a study evaluated the flavonoids extracted through mechanochemical-assisted extraction (MCAE) from pomace of SB [125]. They found that the flavonoids at 26.82 ± 0.53 mg/g exhibited significant beneficial activity in improving liver function.

### 5.10. Neuroprotective Activities

Neuroprotective activity refers to the ability to prevent illness or injury to the brain and nervous system, helping to prevent oxidative stress and protect against free radicals, both of which are toxic mechanisms that can damage brain cells and tissues. In 2021, approximately 3.4 billion people worldwide were affected by or prone to neurodegenerative diseases, accounting for 43.1% of the global population, with 11 million reported deaths [126]. This increased burden has led to a focus on natural remedies that support neural health through mechanistic pathways [127]. Specifically, the active chemical profiles of medicinal plants with the capacity to regulate inflammatory pathways such as Nrf2, and NF-κB are in the focus of research. These pathways play a crucial role in maintaining mental health in response to the negative effects of IL-1β, IL-6, and TNF-α [128].

Alzheimer’s disease is a neurodegenerative disorder characterized by a proliferation of extracellular Aβ and neurofibrillary tangles caused by hyperphosphorylation of the Tau protein. SB powder helps reduce intracellular Aβ plaques. These findings of this study indicate that found that the antioxidants in SB powder inhibit Aβ-induced toxicity and prevent cell death, exhibiting a neuroprotective effect [129]. Previous research has shown that the flavonoids in SB increase the activation of the insulin receptor substrate and AKT, leading to a reduction in protein tyrosine phosphatase 1B (PTP1B) and the recovery of the extracellular signal-regulated kinase (ERK)/cAMP response element binding protein (CREB)/brain-derived neurotrophic factor (BDNF) and insulin signaling pathways. This helps prevent memory loss, alleviate high-fat-diet-induced cognitive injury, and reduce insulin resistance and neuroinflammation [82].

A study investigated the neuroprotective and antioxidant properties of SB’s total flavonoids. The conclusion of the study demonstrated that the TFH could scavenge superoxide anion, hydroxyl radicals, and 2,2-Diphenyl-1-picrylhydrazyl (DPPH) radicals [130]. Monoamine oxidase A (MAO-A) and acetylcholinesterase (AChE) were used to evaluate the neuroprotective potential. 50 µg/mL TFH inhibited AChE and MAO-A at 75.85% and 51.22%, respectively, showing strong radical scavenging activity. It also delayed paralysis in an Alzheimer’s *Caenorhabditis elegans* model, supporting the anti-aging and neuroprotective potential. Another study revealed the effect of SB polysaccharide (SBP) at doses of 100–200 mg/kg for several weeks [131]. The findings of the study indicated that SBP ameliorated HFD-induced cognitive deficits, reduced neuroinflammation and oxidative stress, and improved synaptic plasticity markers.

Similarly, a study revealed lowered neuroinflammation and cognitive improvement by isorhamnetin and SB flavonoids when a dose of approximately 30–60 mg/kg/BW was given to mice [132]. The findings showed that isorhamnetin mitigates neuroinflammation, reduces oxidative stress and microglial activation, and improves cognitive performance in obesity. It also impacts NF-κB and Nrf2 pathways. Likewise, another study reported the use of SB seed oil, nebulized SSO inhalation in APP/PS1 mice for 21 days [133]. The findings of this study indicated improvement in memory and cognition with nebulized SB seed oil, as well as reduced amyloid pathology markers and neuroinflammation in transgenic Alzheimer’s disease.

### 5.11. Cardioprotective Activities

Cardiovascular diseases (CVDs) are the leading cause of mortality, damaging the blood vessels and heart. This category includes various conditions such as coronary heart disease, peripheral artery disease, cerebrovascular disease (e.g., stroke), pulmonary embolism, deep vein thrombosis, and congenital heart defects [18]. Engaging in cardiovascular activities can help lower levels of triglycerides and cholesterol, which are risk factors for heart attacks and atherosclerosis. Additionally, CVDs account for 438 million disabilities, highlighting the need for comprehensive strategies to manage them [134].

Worldwide, CVD is the leading cause of death, responsible for over 17.9 million deaths annually, which is about 32% of all deaths globally. The risk is increasing in middle and low-income countries due to changing lifestyles, unhealthy diets, and limited access to healthcare. Prevention through lifestyle changes and early screening is now a significant global health priority [29]. Engaging in cardiovascular activities may improve cholesterol profiles by increasing high-density lipoprotein (HDL) and reducing LDL oxidation [5]. These activities also support cardiovascular and metabolic health by lowering cholesterol, promoting circulation, having anti-atherogenic effects, and reducing the risk of thrombophlebitis [135].

The risk factors that contribute to CVD include vascular sclerosis, hyperglycemia, hyperlipidemia, myocardial hypertrophy, and hypertension. SB reduces the blood viscosity and enhances flavonoids by scavenging free radicals, thereby improving cardiac function [136]. SB may also help prevent coagulation and hypertension. Another health benefit of SB in relation to CVD is its ability to lower blood sugar levels, blood cholesterol and blood pressure. The significant antioxidant effect of SB is the main reason for improved CVD symptoms, lower lipids and blood pressure, reduced levels of free radicals, and prevention of atheroma [18].

Patients who took 10 mg of flavonoid extract of SB three times per day for six weeks showed reduced cholesterol levels and improved cardiovascular function [137]. The reduction of pro-inflammatory factors by the intake of SB extract may have the effect of reducing myocardial stress. HDL and LDL levels can be raised and lowered in plasma. Consequently, in vivo investigations suggest a reduced risk of CVD [138]. Recent studies, including a systematic review analyzing 15 randomized controlled trials, show that SB supplementation significantly improved lipid profiles by lowering triglycerides, total cholesterol, and LDL-C, while enhancing high-density lipoprotein cholesterol (HDL-C), but only in individuals with abnormal lipid metabolism [60].

**Figure 5:**
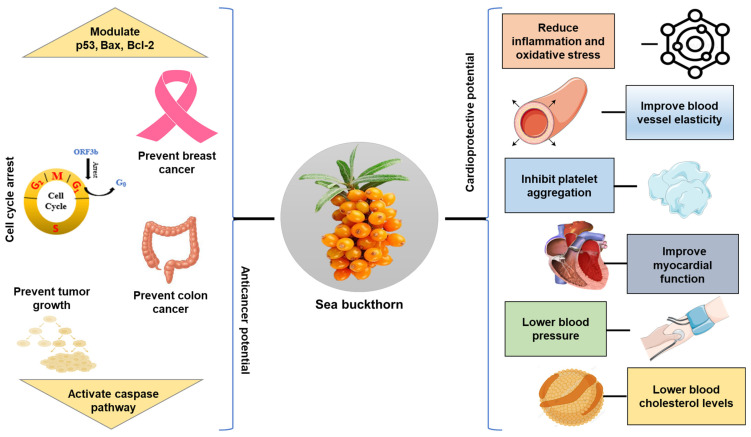
Schematic overview of anticancer and cardioprotective mechanisms of sea buckthorn bioactives, including modulation of cell cycle checkpoints, pro- and anti-apoptotic proteins (p53, Bax, Bcl-2), and signaling pathways that influence oxidative stress, inflammation, lipid metabolism, and vascular function.

Previous studies have shown that SB treatment for 60 days improved left cardiovascular clinical results, cardiovascular function, and plasma nutrition in rats with spontaneously hypertensive stroke. A study demonstrated that vitamin E and provitamins A, among other components such as rutin, serotonin, cytosterol, zinc, and selenium are present in the dry powder of SB berries. These components caused significant reductions in heart rate, arterial blood pressure, triglycerides, glycated cholesterol, and hemoglobin in mice fed chow supplemented with 0.7 g/kg SB powder [139]. Treatment also reduced the alkaline phosphatase-positive microvessels and enhanced the total capillary density. All the results indicate that SB enhanced metabolic health and alleviated the hypertensive load of ventricular microvascular stress (Figure 5).

Another study demonstrated the beneficial effects of orally administering of SB flavone (75 mg/kg/day for 6–12 weeks) in ApoE-deficient mice [129]. The flavone inhibited macrophage foam cell formation, reduced inflammation, and curbed vascular plaque development. Recent studies have shown that SB seed flavones (50–150 mg/kg/day for 8 weeks) can reduce hypertension, insulin resistance, and lipid abnormalities in sucrose-fed hypertensive rats. Another study demonstrated the cardioprotective effects of SB, such as inhibiting platelet aggregation, lowering blood pressure, and reducing cholesterol levels. These effects are largely attributed to the high vitamin content (A, C, E), unsaturated fatty acids, flavonoids, and phytosterols present in SB [130].

### 5.12. Skin-Related Effects

Skin healing is a complex biological process in which the skin regains its structural and functional integrity after injury. The proliferation of tissues and remodeling involve coordinated cellular, humoral, and molecular mechanisms [131]. Recent research indicates a global interest in SB wound healing properties, particularly through the development of advanced topical formulations like nano-emulsion-based creams and gels using SB fruit oil. These formulations aim to enhance skin penetration, epithelization, and overall healing efficiency [132].

Similarly, others demonstrated the photo protective activity of SB fruit pulp extract against UV-B-induced damage on primary human dermal fibroblasts (HDFs) and Balb/c mice’s skin [133]. We exposed both sets of mice to UV-B radiation and evaluated various markers of cellular damage. The findings showed that UV-B irradiation induced ROS, causing endoplasmic reticulum (ER) stress, apoptosis, inflammation, DNA damage, and extracellular matrix degeneration. SB flavonoids stimulate wound healing by enhancing antioxidant properties, promoting wound closure and epithelialization. Additionally, flavonoids increase glutathione, vitamin C, and catalase levels in the wound granulation tissue, leading to reduced lipid peroxidation [134].

The main moisturizing essential fatty acid, and a naturally occurring component of skin, is palmitoleic acid [135]. It provides anti-aging moisturizing benefits for skin and mucous tissues [136]. An interesting 5% aqueous leaf extract of SB (SBTL-ALE) significantly accelerated wound healing in diabetic rats. Findings included faster epithelial closure, increased collagen (hydroxyproline) and hexosamine levels, important antioxidants (SOD, catalase, GSH), and reduced oxidative stress. Mechanistically, it enhanced TGF-β and α-smooth muscle actin expression key healing mediators [137].

SB oil contains anti-inflammatory and anti-psoriatic effects. The high content of fatty acids in SB oil may be the mechanism behind these actions, as it inhibits reactive nitrogen and pro-inflammatory cytokines, as well as NF-κB protein [138]. Palmitoleic acid, an essential therapeutic compound found in SB oil, is beneficial for healing injuries and soothing mucous membranes and tissues. Research has demonstrated that palmitoleic acid can help rejuvenate the skin in the epidermis and aid in repairing cracks. Ingesting SB oil also increases levels of linolenic acid in cell membranes, which can alleviate symptoms of dermatitis [139]. Table 3 illustrates how SB oil plays a key role as a natural lubricant in supporting skin health.

## 6. Safety and Toxicity

Research on the safety and toxicity of SB is still limited, despite its long history of use in nutrition and therapy. Studies on SB berry oil have shown no evidence of genotoxicity, mutagenicity, or carcinogenicity [146]. It does not induce mutations in *Salmonella typhimurium* strains and shows no adverse effects on sperm morphology or micronucleus formation in animal models at high doses. Similarly, no teratogenic or embryotoxic effects were observed in pregnant animals, even at relatively high intake levels. The No Observed Adverse Effect Level (NOAEL) for SB berry oil in rats has been reported at approximately 4.68 g/kg body weight, while aqueous fruit extracts showed a NOAEL of around 100 mg/kg body weight/day in sub-chronic studies [147]. Furthermore, a study revealed an insight into the acute toxicity of SB oil in mice [148]. The highest amount of SB oil that the mice could tolerate was found to be greater than 18.72 g/kg. The NOAEL was determined to be 9.36 g/kg BW based on repeated oral toxicity studies conducted on rats over a 90-day period. SB has been classified as safe and non-toxic for consumption as a dietary supplement, pharmaceutical, or human food.

Acute and sub-chronic toxicity studies further support the safety of SB. High oral doses of SB oil (above 18 g/kg body weight) were well tolerated in animal models, indicating very low acute toxicity. In 90-day repeated-dose studies in rats, no significant changes were observed in body weight, organ weight, food intake, hematological parameters, or histopathological findings [149]. Similarly, SB leaf extracts demonstrated low toxicity, with an LD_50_ greater than 10 g/kg body weight, although minor changes in liver and kidney weights were noted at higher doses [150]. Overall, current evidence suggests that SB and its derivatives (oil, extracts, and leaf preparations) are non-toxic and safe for use as food ingredients, dietary supplements, and therapeutic agents. However, further well-designed long-term and human clinical studies are needed to fully establish their safety profile, particularly for high-dose and prolonged use.

## 7. Sea Buckthorn Encapsulation

Encapsulation is the process of enclosing an active substance (nutrients, bioactive compounds, or drugs) within a protective coating or carrier substance to create tiny capsules. These techniques help protect the core material from environmental factors such as heat, light, or oxygen, improve stability, control release, and enhance the delivery of substances in food, pharmaceutical, or cosmetic applications [151]. The encapsulation food market is rapidly increasing, estimated to be around USD 7.1 billion in 2025, with projections soaring to USD 18 billion by 2035 at a compound annual growth rate of approximately 9.8% [152].

Previous studies have shown that bilayer nanoparticles can be created using zein and gum Arabic to encapsulate SB flavonoids. These nanoparticles achieved an encapsulation efficiency of approximately 77.2%, demonstrated enhanced stability during storage (60.5% retention) and in vitro digestion (53.8% retention). Additionally, they significantly improved cellular absorption and antioxidant activity, including ABTS (85%) and DPPH (80%) radical scavenging, while effectively reducing intracellular reactive oxygen species (ROS) [153].

In a study by Kumar and colleagues, carotenoids (primarily beta-carotene and zeaxanthin) extracted from SB pomace were encapsulated using calcium-alginate hydrogel beads via ionotropic gelation [154]. This superior efficiency suggests that ionotropic gelation is more effective at trapping lipophilic carotenoids within a polymer matrix than traditional thermal methods. Practically, the stability of over 64% for 30 days at both 4 °C and 25 °C indicates that these hydrogel beads are suitable for fortifying refrigerated or room-temperature “wet” food systems, such as yogurts or jams, where oxidative degradation is usually rapid. Furthermore, the moderate bio-accessibility (~42%) represents a significant improvement over non-encapsulated carotenoids, which often struggle with micellization in the digestive tract. While lower than some nano-emulsion systems, this level of bio-accessibility provides a functional balance between cost-effective production and therapeutic delivery, making it a viable strategy for large-scale production of fortified functional foods [155].

Another study by Mo and colleagues revealed that SB seed oil could be spray-dried using maltodextrin and inulin (total wall content 15%, core to wall ratio 1:3, inlet temperature 160 °C) [156]. The findings of the studies indicated that the microcapsules showed excellent encapsulation efficiency, structural stability, and controlled release potential, making them ideal for use in functional foods and nutraceutical formulations.

Similarly, a study demonstrated the effect of spray drying parameters on the physicochemical properties of encapsulated SB berry oil and evaluated the different carriers (gum Arabic, β-cyclodextrin, or a mixture in a 1:1 ratio), inlet air temperatures (120, 150, and 180 °C), and carrier to oil ratios (2,3, and 4) [157]. The investigations indicated the moisture content (0.23–3.70%), encapsulation efficiency (73.08–93.18%), product yield (36.79–64.60%), solubility (19.55–74.70%), hygroscopicity (1.5–7.06 g/100 g), antioxidant capacity (871.83–1454.39 μmol TE/100 g dm), bulk density (0.25–0.44 g/L), and total carotenoid content of the resulting powder were examined. Findings of this study reveal that the antioxidant potential of SB berry oil increased when raising the carrier ratios, improving efficiency, yield, and stability under optimal spray conditions, which helps preserve the antioxidants and carotenoids.

Encapsulating SB transforms it into a robust functional ingredient by protecting its bioactive compounds from heat, light, and oxidation during industrial processing. In food systems, this technology enables the seamless fortification of baked goods and beverages by enhancing solubility and masking the fruit’s intense natural acidity [158]. Techniques like hydrogel beads or liposomes significantly improve the stability of SB in dairy products, ensuring higher bio-accessibility of antioxidants during digestion. Furthermore, SB extracts incorporated into meat products serve as a natural antioxidant, effectively preventing lipid rancidity while potentially replacing synthetic additives [159]. Ultimately, the application of encapsulation technologies ensures the long-term stability and controlled release of SB bioactives, maintaining their functional potency from raw material processing to final metabolic absorption.

## 8. Food Applications for Sea Buckthorn

**Figure 6:**
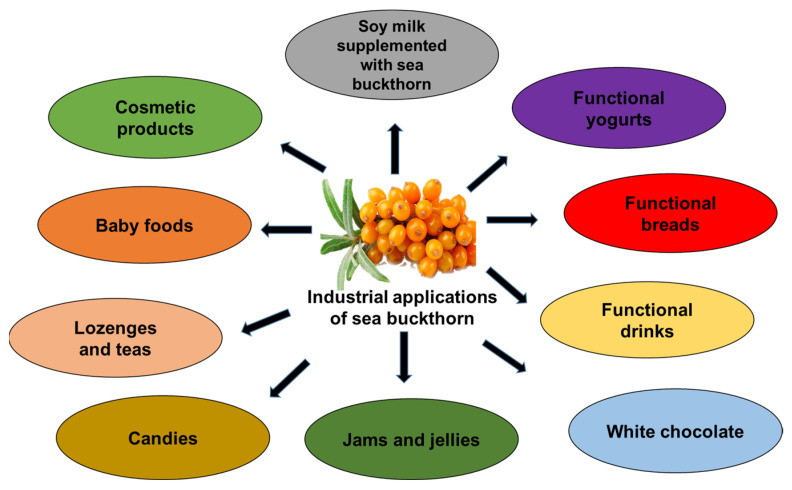
Selected industrial applications of sea buckthorn ingredients in foods and cosmetics, including their use in jams, jellies, functional yogurts, soy beverages, candies, breads, baby foods, functional drinks, lozenges, teas, white chocolate, and topical formulations.

SB contains a variety of bioactive substances and is rich in nutrients. Nowadays, SB is used in numerous food products as a natural ingredient, antioxidant, and antibacterial agent. Culinary uses of SB have expanded in the food industry, with SB oil, freeze-dried powder, fruit juices, milk tablets, tea, fruit vinegar, wine, preserved fruit, jam, and yogurt being some of the products made with SB (Figure 6). The food industry, manufacturers, and researchers are currently exploring the consumption of SB to enhance the nutritional value and sensory qualities of products (Table 4).

### 8.1. Food Additive Industry

SB extracts are widely used as natural antioxidants and preservatives, reducing lipid oxidation and microbial growth in food systems. The meat sector is currently seeking natural alternatives to replace the synthetic additives used in its products [160]. The global SB juice market was valued at approximately USD 347.56 million in 2023 and is projected to reach USD 684.25 million by 2030 with a growth rate of 10.16%. Adding SB fruit ethanolic extract to pork sausages at a rate of 3% prevented lipid oxidation and reduced the total amount of bacteria. The total number of sausage colonies decreased by approximately seven times, enhancing the sausages’ microbiological richness [161]. A functional bread was developed by incorporating 6%, 8%, and 10% SB pomace powder from three organic varieties (Marla, Clara, and Sorana) into wheat flour, improving the nutritional characteristics of the bread. It presented higher antioxidant activity, polyphenolic content and crude fiber [162].

When SB fruit powder is added to wheat bread, the bread’s shelf life increases by one to three days and improves the bread’s antioxidant and organoleptic qualities [159]. SB juice is gaining popularity due to its high vitamin C content, which surpasses that of oranges, and its antioxidant properties [163]. SB from Finland is used as a functional ingredient in baby food. Its medicinal benefits, along with its significant economic importance, are highly valued [164]. Currently, SB is utilized as a natural antioxidant and antimicrobial agent, enhancing various food products [165]. Foods like fermented juices, milk-based lozenges, vinegar-based beverages, teas, candied fruits, yogurts, and preserves are increasingly incorporating SB in the food industry [166]. The study also revealed antimicrobial properties against *Pseudomonas aeruginosa*, *Bacillus cereus*, *Klebsiella pneumonia*, *Methicillin-resistant Staphylococcus aureus* (*MRSA*), and *Salmonella enterica*. Recent studies report the development of SB-based powdered additives for improving the nutritional and functional properties of foods [167].

### 8.2. Dairy Industry

As a novel plant-based additive, SB is gaining popularity in dairy production worldwide due to its healthful, nutritional benefits. It has gained prominence in dairy production all over the world because of its health-promoting qualities. The addition of SB to yogurt enhances its nutritional value as it is a rich source of nutritionally active compounds. SB yogurt contributes to dietary intake of carbohydrates, fat, protein, and antioxidants. SB yogurt maintains its microbiological quality even after being refrigerated at 4 °C for 12 days and at 15 °C for 3 days [168]. Adding a variety of ingredients and flavors from vegetables and fruit helps compensate for nutritional deficiencies [169]. Fermentation of SB juice or meal improves the flavor, reduces oiliness, increases metabolites, and influences gut microbiota. SB puree has been added to dairy products such as kefir, yogurt, and cheese, enhancing their antioxidant properties and organoleptic qualities [165]. SB supplementation in yogurt (0–20 percent) and 10 percent formulation was found to have the most agreeable composition, with vitamin C (20.65 mg/g), carotenoids (1.46 mg/g), vitamin E (12.00 mg/g), and antioxidant activity (85.1%) [170]. Moreover, fortification of yogurt with lyophilized extracts of SB significantly enhanced water-holding power, decreased syneresis, and overall phenolic content with 0.5–1% having optimal sensory acceptance and increased physicochemical properties of yogurt [171]. The addition of SB in soymilk (20% syrup or 3% powder), inulin (1–3%), and *Lactobacillus casei* reported to improve probiotic viability, stability during 14 days’ storage, and improved sensory quality [172].

### 8.3. Confectionery Industry

The SB fruit has a short shelf life and a bitter flavor. Making jam from these berries is a good way to enhance their flavor and increase their utility. Jam made from SB fruits at 102 °C with stevia has a high concentration of polyphenols and carotenoids, revealing antioxidant characteristics. SB by-products, when used as a functional ingredient in white chocolate, have shown better texture, antioxidant activity, and vitamin and mineral content, particularly at a 15% rate [173]. The functional jelly candies, created by adding up to 4% dried pomace of SB to a quince puree foundation, enhanced antioxidant activity by 91.71% in terms of DPPH radical scavenging activity, with a total polyphenol content of up to 633.28 mg GAE/100 g. Although the fiber content was high (6.61% to 7.19%), sensory analysis revealed that 1% and 2% SB additions were the most acceptable due to their balanced composition of taste and texture [174]. Additionally, a functional jam was developed using SB berries, sesame milk and honey, which improved moisture (23.0 g/100 g), protein (3.8 g/100 g), vitamin C (82.21 mg/100 g), calcium (118.2 mg/100 g), iron (3.26 mg/100 g), zinc (1.8 mg/100 g), and magnesium (16.4 mg/100 g), showing improved nutritional composition over traditional jam [175]. After 21 days of storage at room temperature, less than 100 colony-forming units (CFU)/g of yeast and mold, and less than 5 CFU/g of *Enterobacteriaceae* have been identified [168]. SB can be combined with sweet potatoes, carrots, and pumpkins in specific amounts to create a new, nutritious jam [176]. The shelf life of the jam is 177 days at 20 °C without the addition of preservatives. Moreover, the combination of SB with carrots, pumpkins, and sweet potatoes in a minimal ratio can create a nutritious and innovative jam [177]. Mixing SB juice with other fruit juices such as watermelon or grape, can create a delicious and healthy jam. SB contains a variety of bioactive substances suitable for sugar-based products. Cookies made from SB dried powder have shown increased polyphenols and antioxidant activity. Sensory changes such as taste, color, and texture also improve with higher inclusion levels [178].

### 8.4. Beverages Industry

The SB processing industry generates waste from the berries of SB, and improper disposal of this waste pollutes the environment. Research by Lele and colleagues demonstrated that using byproducts of SB juice in chewing gum formulations can significantly enhance antioxidant activity [179]. Fermentation can be conducted on the waste from the SB processing sector. Under optimal fermentation conditions, SB beverages with 3% ethanol were produced. This beverage is a pleasant and beneficial healthy drink because it contains high levels of phenolic compounds, such as gallic acid, chlorogenic acid and vanillic acid, and has a strong antioxidant effect. It also has low levels of ethanol and carbon dioxide [180]. The process of micro-wet milling can be utilized to produce fruit pulp juice from SB. The result of this process produces a bright yellow color and improves total carotenoid concentration, vitamin C levels, total phenolic content, and antioxidant activity. This approach prevents the degradation of biologically active molecules that are sensitive to heat. Additionally, it produces fiber-rich juice that has great potential for use in the SB juice processing industry [181].

The SB drink sweetened with monk fruit and processed with the LAB cultures (*L. plantarum*, *L. acidophilus*, *L. paracasei*) also increased phenolics, antioxidant activity, and sensory attributes and improved the flavor of the SB drink [182]. Moreover, fermented SB juice with probiotics, increased the bioactive composition and functionality of the juice and enhanced SOD activity (725.44 U/mL), total flavonoids (2.38 mg/mL), and antimicrobial and antioxidant activity [183]. The incorporation of SB powder (2–12%) in apricot probiotic drink with *Lactobacillus rhamnosus*. The 4% formulation showed optimal composition with the highest probiotic count (6.7 log CFU/mL) and improved total phenolics, antioxidant activities and functional properties [184].

### 8.5. Cosmetics Industry

SB is a potent source of vitamin C, which aids in collagen production and protects the skin from UV damage [185]. Its unsaturated fatty acids also assist in skin repair and healing [132], enhance the activity of specific enzymes (e.g., MMPs), boost collagen production, and promote wound healing by removing damaged proteins and supporting tissue growth [186]. These benefits underscore the significance of SB in the cosmetics industry, with creams and shampoos being the primary SB-based products available [33].

SB is utilized for encapsulation, protective coating, and as a carrier substance to create small capsules. These techniques safeguard the core material from environmental factors like heat, light, or oxygen, enhance stability, control release, and improve the delivery of substances in food, pharmaceutical, or cosmetic applications [144]. The encapsulation food market is rapidly growing, reaching USD 7.1 billion in 2025, with projections soaring to USD 18 billion by 2035 at a compound annual growth rate of around 9.8% [151]. Bilayer nanoparticles using zein and Arabic gum to encapsulate SB flavonoids have been shown to enhance encapsulation efficiency up to 77.2%, stability during storage (60.5% retention), and in vitro digestion (53.8% retention). Additionally, they significantly improved cellular absorption and antioxidant activity, including ABTS (85%) and DPPH (80%) radical scavenging, while effectively reducing intracellular ROS [152].

As discussed above, a study by Kumar and colleagues successfully encapsulated carotenoids (primarily β-carotene and zeaxanthin) extracted from SB pomace using calcium-alginate hydrogel beads via ionotropic gelation [153]. The results demonstrated an impressive 98.4% encapsulation efficiency, stability of over 64% for at least 30 days at both 4 °C and 25 °C, and moderate bio accessibility (~42%) in simulated digestion systems. Another study by Mo and colleagues showed that SB seed oil could be spray-dried using maltodextrin and inulin (total wall content 15%, core to wall ratio 1:3, inlet temperature 160 °C) [155]. The findings indicated excellent encapsulation efficiency, structural stability, and controlled release potential, making them suitable for use in functional foods and nutraceutical formulations.

A previous study suggested that liposomal systems loaded with both lipophilic SB extracts and hydrophilic grape pomace components achieved high encapsulation efficiency (~84–90%) and retention rates (~79–86%) [154]. The study revealed that the encapsulated extracts exhibited higher antioxidant activity during gastric digestion compared to non-encapsulated ones, although the activity decreased over time under intestinal (alkaline) conditions. Similarly, another study examined the effect of spray drying parameters on the physicochemical properties of encapsulated SB berry oil. Different carriers (gum Arabic, β-cyclodextrin, or a 1:1 mixture), inlet air temperatures, and carrier to oil ratios were evaluated [150]. The investigations focused on moisture content, encapsulation efficiency, product yield, solubility, hygroscopicity, antioxidant capacity, bulk density, and total carotenoid content of the resulting powder. The findings of this study revealed that the antioxidant potential of SB berry oil increased when raising the carrier ratios. This improvement enhanced encapsulation efficiency, yield, and stability under optimal spray conditions, ultimately helping to preserve the antioxidants and carotenoids.

## 9. Limitations and Future Perspectives

Despite the promising pharmacological and nutritional value of SB, the current evidence base is still limited. Most studies are small, heterogeneous in design, and use non-standardized preparations, which hampers comparison between studies and translation into practice. A major challenge is the lack of standardized formulations; extracts differ widely in their content of key constituents such as flavonoids, vitamins, and omega fatty acids. Moreover, the bioavailability, metabolism, and pharmacokinetics of these bioactives in humans are insufficiently characterized, so appropriate dose ranges for prevention or therapy remain unclear. While in vitro and animal data support antioxidant, anti-inflammatory, cardioprotective, neuroprotective, and other effects, robust clinical trials in humans are scarce, especially for chronic conditions such as metabolic syndrome, asthma, neurodegenerative diseases, and dermatological disorders. Thus, although the diverse SB phytochemical profile is attractive for developing pharmaceuticals and functional foods, standardized extracts, well-described bioavailability and pharmacokinetics, clearly defined effective doses, and long-term safety data are still needed.

From a food technology perspective, the use of SB ingredients also faces practical formulation constraints. Pronounced acidity, astringency, and a characteristic aroma restrict feasible inclusion levels and often necessitate blending with other fruits, sweeteners, or flavor-masking strategies to achieve acceptable sensory profiles. Furthermore, the stability of vitamin C, carotenoids, phenolics, and unsaturated fatty acids during processing and storage strongly depends on the food matrix, processing conditions (e.g., heat, oxygen exposure, light), and packaging, which have not been systematically evaluated across major product categories. Interactions with matrix components (such as dairy proteins, starches, or emulsifiers) may affect color, texture, and bioaccessibility, underscoring the need for more systematic formulation, process, and shelf-life studies under industrially relevant conditions.

Future research on SB in food systems should integrate sensory and consumer acceptance studies with mechanistic work on bioaccessibility and bioavailability in specific matrices, including dairy, baked goods, beverages, and meat products. Advanced delivery approaches, such as encapsulation, structured emulsions, and valorization of by-products (pomace, leaves, twigs), offer promising routes to improve stability and controlled release of bioactives while supporting circular-economy concepts in the food industry. In parallel, techno-economic analyses and regulatory assessments are required to define realistic enrichment levels, labeling possibilities (e.g., “functional” or “superfruit”), and safety margins for long-term consumption of SB-enriched foods at scale.

Overall, it will be crucial to develop nutritionally compatible, bioavailable SB formulations and test them in well-designed human trials, while advances in extraction technologies and nutrigenomics may clarify how SB can best support individual health needs.

## 10. Conclusions

The analysis of metadata from the previous literature has highlighted SB and its bioactive profile, which is highly valuable due to its numerous medicinal attributes. These attributes include antioxidant, dermatological, cardioprotective, hepatoprotective, neuroprotective, antiviral, antimicrobial, anti-inflammatory, and anti-asthmatic properties. SB is rich in essential fatty acids, carotenoids, vitamins, especially vitamin C, polyphenols, and phytosterols. In addition to its therapeutic benefits, SB is increasingly being incorporated into food products to enhance their physicochemical and rheological properties, extend shelf life, and boost nutritional value. However, further in vitro and in vivo studies are needed to clarify dose-dependent efficacy and to establish robust placebo-controlled clinical trials for safe translational use. Undoubtedly, SB has significant potential as an ingredient in developing functional foods and supplements due to its rich and nutritious composition, but advanced research is needed to fully realize its potential in these sectors and support food security.

## Figures and Tables

**Table 1:** Representative concentrations of macronutrients, micronutrients, minerals, and bioactive compounds in different anatomical parts of sea buckthorn ^1^.

Plant Part	Parameter	Representative Value (Unit, Basis)	Notes on Composition/Source
Berries (whole fruit/pulp) [31,32,33,34,35,36]	Energy	~79 kcal/100 g FW	From proximate analysis of fresh berries
	Protein	3.1 g/100 g FW	Includes all essential amino acids: His, Leu, Ile, Lys, Phe, Thr, Val each ≈0.2–0.4 g/100 g FW
	Fat	2.5 g/100 g FW	Mixed saturated/unsaturated lipids
	Total sugars	4.5 g/100 g FW	Mainly glucose and fructose (in juice: 0.6–24.2 g/100 mL depending on variety)
	Fiber	6.55 g/100 g FW	Includes pectin and other non-starch polysaccharides
	Vitamin C	170–695 mg/100 g FW (typical range)	170 mg/100 g FW in Iranian wild berries, ~275 mg/100 g FW in Himalayan pulp, upper values up to ≈2500 mg/100 g reported in some cultivars
	Vitamin A (as β-carotene)	~20 mg/100 g FW (β-carotene), 432 IU/100 g vitamin A	0.20 mg/g FW β-Carotine in Iranian Berris
	Vitamin E	~3.5 mg/100 g FW	Measured in Himalayan pulp
	B-vitamins	Riboflavin 1.45 mg, niacin 68.4 mg, B6 1.12 mg, pantothenic acid 0.85 mg/100 g FW	Representative pulp composition
	Potassium	650–790 mg/100 g FW	Potassium is the dominant mineral in fruit
	Other minerals	Ca 110–129 mg, Mg ≈ 99 mg, P 49–111 mg, Na ≈ 155 mg, Fe 29–31 mg, Zn ≈ 3 mg, Mn ≈ 1 mg/100 g FW	Representative ranges from different origins
	Total phenolics	200–250 mg GAE/100 g FW	Example: 247 mg GAE/100 g FW in Iranian berries
	Anthocyanins	3–7 mg/L juice	Cyanidin-3-O-glucoside equivalents
	Carotenoids (total)	3–15 mg/100 g FW	Mainly β-carotene, zeaxanthin, lycopene
	Representative fatty acid profile (fruit oil)	Linoleic 34.2%, palmitoleic 21.4%, palmitic 17.2%, oleic 12.8%, α-linolenic 5.4%, stearic 1.7% of total fatty acid	Typical berry lipid profile
Seeds (whole and seed oil) [32]	Protein	20–25% of seed DM	Seed meal rich in storage proteins (globulins)
	Seed oil content	8–20% of seed mass	Range across cultivars and locations
	Fatty acids (seed oil)	Linoleic 35–42%, α-linolenic 20–35%, oleic 15–21%, palmitic 6–10%, stearic 2–3% of total FA	Example: linoleic 42.36%, linolenic 21.27%, oleic 21.34%, palmitic 6.54%, stearic 2.54%
	Vitamin E and K	≈207 mg vitamin E/100 g oil, 110–230 mg vitamin K/100 g oil	Together with 100–200 mg/100 g tocopherols and tocotrienols
	Carotenoids and sterols	Carotenoids 10–50 mg/100 g oil, plant sterols 1–2%	β-sitosterol and related phytosterols predominate
Leaves [37,38,39,40]	Crude protein	18–21 g/100 g DM	Range 15.7–20.8 g/100 g DM across years/cultivars
	Ether extract (fat)	5–6.9 g/100 g DM	Lower in Ascola, higher in Habego/Hergo
	Crude fiber	9.6–12.1 g/100 g DM	NDF 21–32 g/100 g DM, cellulose 8.7–14.6 g/100 g DM
	Vitamin C	up to 75 mg/100 g FW	Reported in some leaf materials
	B-vitamins and vitamin E	Niacin 31–46 mg, pyridoxine 33–81 mg, vitamin E 9–26 mg, riboflavin up to ≈230 mg/100 g DM	Russian leaf samples (dry basis)
	Major minerals	Ca 12–24 g/kg, K 7–12 g/kg, Mg ≈ 0.7–2.8 g/kg, P ≈ 1.1 g/kg DW	Plus Fe ≈ 9 g/kg, Zn ≈ 2.5 mg/kg, Na ≈ 13.9 g/kg
	Total carotenoids and chlorophyll	Carotenoids ≈ 1004 mg/kg DW, chlorophyll ≈ 988 mg/kg DW	Equivalent to ≈100 mg and 99 mg/100 g DW
	Total phenolics	≈187–330 mg GAE/g DM	Very high polyphenol density, cultivar and year dependent
	Total flavonoids and flavonols	≈282 mg quercetin eq./g DM (TF), ≈80 mg quercetin eq./g DM (flavonols)	Sea buckthorn leaf extracts rich in flavonols (rutin, quercetin)
	Proanthocyanidins and tannins	≈7.9–18.5 mg/g DM PAC, ≈1.4–1.5 mg TA eq./g DM tannins	Condensed tannins (e.g., catechin/epicatechin units) common
	Selected individual compounds	Rutoside ≈ 0.28–0.31 µg/g DM, quercetin ≈ 0.19–0.22 µg/g, delphinidin ≈ 0.72–1.03 µg/g, chelerythrine ≈ 0.68–1.04 µg/g	HPTLC of leaf extracts
Pulp oil (fruit soft parts) [32,41]	Fatty acids	Palmitoleic 15–50%, palmitic 15–40%, oleic 10–20%, linoleic 5–15%, α-linolenic 5–10% of total FA	High omega-7 palmitoleic acid is distinctive
	Vitamin E and K	~171 mg vitamin E/100 g oil, 54–59 mg vitamin K/100 g oil	With 100–400 mg/100 g total tocopherols/tocotrienols
	Carotenoids and sterols	100–400 mg carotenoids/100 g oil, plant sterols 2–3%	Responsible for intense orange color and antioxidant activity
Branches (wood and bark) [38,42]	Protein	≈9–13% of DM	Russian branches from several cultivars
	Carbohydrates and fat	Soluble carbohydrates 3.15–4.66% DM, fat ≈0.6–1.0% DM	More structural than storage
	Crude fiber	Often >15–20% of DM	Mainly cellulose, hemicellulose, lignin
	Vitamins	Niacin 31–46 mg, pyridoxine 33–81 mg, vitamin E 9–26 mg, riboflavin 1.27–287.54 mg/100 g DM	Substantial B-vitamin content even in woody tissues
	Minerals	Cr 0.38–1.18, Mn 5.16–944, Fe 9.64–42.3, Cu 2.29–4.59, Zn 5.05–19.1, Ni 0.24–2.13, Sr 3.64–59.25 mg/kg, total P 228–1232 mg/kg	Differences between Russian and German material
	Total phenolics	≈0.93–2.50 g/100 g DM	Higher in bark than in wood
	Tannins and other bioactives	High in condensed tannins (incl. hippophaenin A and B), proanthocyanidins, phenolic acids, lignans	Contribute to antioxidant and anticoagulant potential
Twigs [42]	Structural carbohydrates and fiber	High fiber, mainly cellulose, hemicellulose and lignin	Twigs form a significant structural biomass fraction
	Protein and amino acids	Low soluble protein, essential amino acids ≈63% of total, arginine ≈0.87 g/100 g DW	Comparable EAA proportion to leaves
	Minerals	Mainly K and P with smaller amounts of Fe and other trace metals	Similar mineral pattern to branches but less characterized quantitatively
	Phenolics	Butanolic twig extracts up to ≈621 mg phenolics/g dry extract (vs. ≈341.5 mg/g in leaf extracts)	Very high phenolic density, catechins and procyanidins dominate
	Other bioactives	Phenolic acids, lignans, triterpenes, tannins	Implicated in antioxidant and anticoagulant activities

^1^ Data was taken from references [31,32,33,34,35,36,37,38,39,40,41,42]. Abbreviations used are: DM, dry matter; FA, fatty acids; FW, fresh weight; GAE, gallic acid equivalents; NDF, neutral-detergent fiber; PAC, proanthocyanidins; TA, tannic acid.

**Table 3:** Sea buckthorn and skin healing.

Study	Dose/Formulation	Findings	References
Atopic dermatitis (human dietary supplementation)	5 g/day of pulp seed oil or pulp oil vs. paraffin oil, taken orally for 4 months by patients with atopic dermatitis	Pulp oil significantly improved dermatitis scores, while seed oil showed no significant improvement; seed oil also increased plasma alpha-linolenic acid, etc.	[140]
Topical SBT oil in mouse AD-like skin lesions	Daily topical application of SB oil on 1-Chloro-2,4-dinitrobenzene (DNCB)-treated mice for 4 weeks	Through the regulation of NF-kB and STAT1 signaling pathways, SBT oil lowered Th2 chemokines (TARCMDC) and decreased the intensity of dermatitis, epidermal thickness, and mast cell infiltration.	[141]
UV protection in human skin cells	SB seed oil at approximately 500 ng/mL applied to keratinocytes/fibroblasts exposed to UVA (30 J/cm^2^/20 J/cm^2^) or UVB (60/200 mJ/cm^2^)	The oil partially prevented UV-induced ROS generation, enhanced antioxidant levels (GSH, thioredoxin, vitamins A and E), lowered lipid peroxidation, and preserved lipid metabolism; it also induced Nrf2 activity.	[142]
UVB-induced photoaging in human dermal fibroblasts	Alcoholic seed extract (SBSE) of SB seed, tested at 2.5, 5, 10 µg/mL, after UVB irradiation	SBSE increased cell viability, inhibited expression of IL-1β, IL-6, and cyclooxygenase 2 (COX-2); reduced MMP-1 and increased procollagen synthesis, suggesting protection against photoaging.	[143]
Psoriasis treatment in humans	SB extract applied topically (in a single-blind, placebo-controlled study) over treated lesions for 8 weeks	Patients treated with SBT extract showed significant improvement in the Dermatology Life Quality Index (DLQI) and the Psoriasis Area Severity Index (PASI) compared to baseline; placebo showed no benefit at 4 weeks, worsening at 8 weeks.	[144]
Sebum secretion control in healthy volunteers	Topical cream (water-in-oil emulsion) containing 1% concentrated fruit extract administered for eight weeks to volunteer’s cheeks	The formulation reduced sebum secretion compared to the base control; skin stability and physical parameters were stable, indicating possible oil control/cosmetic benefit	[145]

**Table 4:** Selected applications of sea buckthorn in food products ^1^.

Food Category/ Product	Sea Buckthorn Ingredient/ Form	Main Technological/ Functional Role	Key Outcomes (Examples)
Pork sausages	Fruit ethanolic extract (~3%)	Natural antioxidant and antimicrobial properties	Reduced lipid oxidation and a 7-fold decrease in total bacterial count resulted in improved shelf life and microbiological quality
Wheat bread	Pomace powder (6–10%)	Fiber and polyphenol enrichment	Higher antioxidant activity, polyphenol content and crude fiber led to an improved nutritional profile
	Fruit powder	Natural antioxidant and shelf-life extender	Shelf life was extended by 1–3 days with improved antioxidant status and organoleptic quality
Baby foods	Berry juice/puree	Vitamin C and bioactive enrichment	Used as functional ingredient in commercial baby foods due to its high vitamin C and antioxidant capacity
Set yogurt	Pulp/puree (up to ~20%)	Antioxidant, vitamin enrichment, and flavoring	Increased vitamin C, carotenoids, vitamin E and antioxidant activity, increasing sensory quality, especially around 10% addition
Yogurt with extracts	Lyophilized sea buckthorn extracts (0.5–1%)	Phenolic enrichment and texture modifier	Higher total phenolics, antioxidant activity, improved water-holding capacity, and reduced syneresis
Soymilk probiotic drink	Sea buckthorn syrup (20%) or powder (3%) + inulin	Prebiotic/probiotic support and flavoring	Improved probiotic viability and stability during storage, as well as enhanced sensory acceptance
White chocolate	Berry by-products/pomace (up to 15%)	Functional “superfruit” inclusion and antioxidant enrichment	Increased antioxidant activity, vitamin and mineral content, and acceptable texture/sensory quality
Jelly candies	Dried pomace (1–4%)	Functional fiber and polyphenol source	Strong increase in antioxidant activity and total polyphenols with best sensory acceptance at 1–2%
Jams/spreads	Whole berries, puree, pomace with other fruits/vegetables	Functional fruit base and vitamin and mineral enrichment	Higher vitamin C, protein and minerals (e.g., Ca, Fe, Zn) compared with traditional jams increasing microbiological stability
Shortbread cookies	Dried fruit powder	Antioxidant and color/flavor enhancer	Increased polyphenol content and antioxidant activity led to improved sensory parameters at appropriate inclusion levels
Chewing candy/gum	Juice and processing by-products	Antioxidant fortification	Significantly enhanced antioxidant activity was observed in chewing gum formulations
Fermented low-alcohol beverage	Juice and processing waste	Fermented functional drink base	Beverage (approximately 3% ethanol) with high phenolic content and antioxidant capacity, low ethanol and CO_2_
Fruit juice/nectar	Micro-wet milled pulp/fiber-rich juice	High-value juice base and retention of bioactives	Improved carotenoid, vitamin C and total phenolic content resulted in good antioxidant activity with minimal thermal degradation
Fermented functional drink	Monk fruit–sweetened sea buckthorn juice + Lactic acidic bacteria cultures	Probiotic/functional beverage and antidiabetic potential	Increased phenolics, antioxidant activity and improved antidiabetic/antihypertensive potential and sensory attributes were noted
Probiotic sea buckthorn juice	Juice fermented with probiotics	Antioxidant and antimicrobial beverage	Higher superoxide dismutase activity, total flavonoids, antioxidant and antimicrobial activity were observed versus non-fermented juice
Apricot probiotic drink	Sea buckthorn powder (2–12%)	Prebiotic/antioxidant fortification	Optimal at 4%: highest probiotic counts, increased total phenolics and antioxidant activity, and improved functional properties

^1^ For details about listed applications refer to the text.

## Data Availability

No new data were created or analyzed in this study. Data sharing is not applicable to this article.

## Abbreviations

Aβ	Amyloid-beta
BW	Body weight
CFU	Colony forming units
COPD	Chronic obstructive pulmonary disease
CVD(s)	Cardiovascular disease(s)
DILI	Drug-induced liver injury
ER	Endoplasmic reticulum
ERK	Extracellular signal-regulated kinase
GAE	Gallic acid equivalents
HDL	High-density lipoprotein
HDL-C	High-density lipoprotein cholesterol
LDL	Low-density lipoprotein
LDL-C	Low-density lipoprotein cholesterol
NAFLD	Non-alcoholic fatty liver disease
NOAEL	No observed adverse effect level
NF-κB	Nuclear factor kappa B
Nrf2	Nuclear factor erythroid 2–related factor 2
PPARγ	Peroxisome proliferation-activated receptor-γ
ROS	Reactive oxygen species
SB	Sea buckthorn
SBF	Sea buckthorn flavonoid crude extract
SBO	Sea buckthorn berry seed oil
SBFO	Sea buckthorn fruit oil
SBP	Sea buckthorn polysaccharide
SBSE	Sea buckthorn seed extract
UVB	Ultraviolet B
